# Study on the Effect of Core-Shell Abaca Vascular Carriers on the Self-Healing and Mechanical Properties of Thermoset Panels

**DOI:** 10.3390/polym15102245

**Published:** 2023-05-09

**Authors:** K. Venkata Chalapathi, M. N. Prabhakar, Jung-il Song

**Affiliations:** 1 Department of Smart Manufacturing Engineering, Changwon National University, 20 Changwondaehak-ro, Uichang-gu, Changwon 51140, Republic of Korea; 2 Research Institute of Mechatronics, Department of Mechanical Engineering, Changwon National University, 20 Changwondaehak-ro, Uichang-gu, Changwon 51140, Republic of Korea; 3 Department of Mechanical Engineering, Changwon National University, 20 Changwondaehak-ro, Uichang-gu, Changwon 51140, Republic of Korea

**Keywords:** abaca lumens, vinyl ester, self-healing, mechanical properties

## Abstract

Self-healing panels were prepared using vinyl ester (VE) and vascular abaca fibers (unidirectional) through the hand lay-up process. Initially, two sets of abaca fibers (AF) were prepared by filling the healing resin VE and hardener and stacking both core-filled unidirectional fibers in a 90° direction to obtain sufficient healing. The experimental results demonstrated that the healing efficiency increased by approximately 3%. SEM-EDX analysis further confirmed the healing process by exhibiting spill-out resin and the respective fibers’ major chemical elements at the damaged site after self-healing. The tensile, flexural, and Izod impact strengths of self-healing panels indicated improved strengths of 7.85%, 49.43%, and 53.84%, respectively, compared with fibers with empty lumen-reinforced VE panels due to the presence of a core and interfacial bonding between the reinforcement and matrix. Overall, the study proved that abaca lumens could effectively serve as healing carriers for thermoset resin panels.

## 1. Introduction

One of the primary reasons that polymer composite (PC) systems deteriorate is cracking [1]. Vascular networks in self-healing composites (SHCs) show excellent promise in relation to autonomous self-healing. In the current study we looked into the use of natural fiber lumens as self-healing carriers. No research has been conducted to date on how natural fiber lumen shapes affect the characteristics of vascular-based self-healing polymer composites [2]. It can be difficult to detect damage at this extremely small scale. In order to overcome these difficulties, various composites have been investigated [3,4].

High-cost self-healing carriers, inspired by biology, have repaired fracture damage in structural materials successfully without exposure, examination, or human intervention [5,6,7]. Using self-healing systems in composites is critical because they allow the delivery of healing agents to the damaged zone at the proper moment. Intrinsic and extrinsic [8,9] processes are two types of self-healing systems that notably engage in the healing mechanism in composites. Healing carriers must be fragile in order to support proper healing when damage occurs. The adoption of healing systems based on their application and interaction with the main reinforcement in the composite has been generally considered. Three types of healing carrier systems have contributed significantly to the development self-healing: nanocarriers, microcarriers, and macrocarriers [10,11,12]. These healing carriers have been prepared by utilizing a variety of reported methods, including electrospinning to create core-shell nanofiber carriers, microemulsion methods to create core-shell microspheres, and employing widely accessible synthetic hollow fibers [13,14,15]. Vascular networks can be used to transfer a large amount of resin inside the core and repair damage in multiple cracking cycles [16]. Furthermore, the reinforcing vascular networks of the composite serve as a supplementary reinforcement. However, the interaction between composite reinforcements and vascular systems is complicated. In contrast to microspheres, vascular network systems have greater lengths, preventing fracture propagation in composites [17].

Generally, composite failures are controlled by means of crack pinning [18], deflection [19], bowing [20], and bridging mechanisms [21]. Considering crack-controlling mechanisms, selecting healing carriers for self-healing applications is essential in polymer composites. Thermoplastic healing carriers and synthetic carrier systems have considerable disadvantages on composite interfaces [22]. Since synthetic healing carrier systems have a smooth surface, there is insufficient contact with the primary reinforcement in these composites. Multiple healing cycles require the development and production of synthetic vascular carrier-reinforced self-healing composites, which present considerable hurdles. Vascular repair mechanisms play a crucial role in limiting damage and extending the useful life of composites. Notably, embedding natural fiber vascular systems in polymer composites has a significant advantage in that they considerably enhance the fiber–matrix interaction and strength of the composites. Natural fiber lumen systems are beneficial for multiple healing cycles [23] owing to their structural lumen integrity. In addition, using natural fiber carriers significantly affects the balancing of the bio-ecological system [24,25]. Owing to their greater length compared with other healing composites (HCs), these vascular systems can bypass the crack and return it to its normal path along the vascular direction, significantly reducing composite damage. Various researchers have recently promoted self-healing in polymer composites by employing synthetic vascular networks. However, several fiber–matrix interaction problems have been studied in the implantation of artificial vascular systems [26,27,28]. Because of the hydrophobic surface of the synthetic carriers used to reinforce the gap between the fibers, the reinforced composite surface has been found to be uneven and to have poor compatibility [29]. The discharge of healing agents is significantly hindered by the use of synthetic glass/3D printed vascular networks. Additionally, using synthetic HCs significantly affects the deterioration of composites.

The regions of the Philippines, Ecuador, and Costa Rica are rich in abaca fibers. These fibers rank among the strongest natural fibers available on the market. Sheaths made of abaca fibers contain a sizable number of 3–4 m long strands. Each abaca fiber contains between 250 and 400 lumens, as well as a protective covering that serves as a cell wall next to the lumens. Lumens can carry healing agents while preserving fiber properties. The fiber is composed of two layers: a primary layer and a secondary layer that protects the lumen structure. The lumen core is made up of a thin xylem layer that helps to store healing materials inside it and distributes them to the damaged zone via capillary action. Each abaca fibril is tightly bound with hydroxyl groups in the crystalline region. Liu et al. [30] reported that abaca fibers resist rotting and have a specific bending strength, comparable to that of synthetic glass fiber; consequently, they are typically utilized as raw materials for ropes, bags, and paper. In addition, abaca fiber is used as a filler material for bolster and interior trim parts in the automobile sector. Mercedes-Benz body parts are composed of a combination of polypropylene thermoplastic and abaca yarn [31]. Natural fibers can serve as a substitute for glass fibers in automobile components, reduce weight, and provide more environmentally responsible production and recycling processes [32].

In the present study, a novel plant-based bio vascular lumen was used to replace synthetic carriers in polymer composites. The vacuum-assisted resin infusion molding (VARIM) method was adopted to infuse the healing agents (epoxy and hardener) inside the unidirectional abaca fiber lumens. Confirmation of the core was performed using scanning electron microscopy (SEM). The percentage of the core inside the fiber lumens was analyzed. Fiber degradation was analyzed using thermogravimetric analysis (TGA) under a nitrogen atmosphere. The self-healing phenomena exhibited by the healing-agent-embedded abaca fiber/vinyl ester (AF/VE) panels were studied by creating a crack on the surface and side edge using a sharp needle. SEM with energy dispersive X-ray (SEM-EDX) mapping was conducted on the healed zone to confirm the effects of the chemical compounds on the cracked zone. Additionally, a coupled low-velocity impact and tensile test was conducted to evaluate the mechanical and self-healing properties of the AF/VE panel. Moreover, healing was confirmed via SEM analysis. The load-bearing capacity and observed energy capacity of the pure and healing-core-embedded AF/VE panels were tested with three-point bending and Izod impact tests. This study provides a reference regarding the feasibility of using abaca vascular lumens for self-healing applications.

## 2. Materials and Methods

### 2.1. Materials

Unidirectional abaca fibers (with a density and average diameter of 1.5 g/cm^3^ and 0.39 mm) were obtained from Soo industries Co., Gyeongju, Republic of Korea. The healing materials—Diglycidyl ether of bisphenol A (DGEBA (KFR-120V)) (healing resin), Cyclohexanemethanamine, 5-amino-1,3,3-trimethyl (KFH-163) (catalyst), vinyl ester (KRF-1031, Viscosity 150 cps, specific gravity = 1.03), cobalt naphthalate (CN) (promoter), and methyl ethyl ketone peroxide (MEKP-BUTANOX M-60) (catalyst)—were purchased from KUKDO Chemicals, Seoul, Republic of Korea. Double-sided sealant tape was purchased from general sealants Inc., JET Korea, Pocheon, Republic of Korea. Silicone rubber adhesive (Sil-Poxy) was received from Smooth-On, Inc., Seoul, Republic of Korea.

### 2.2. Manufacturing of Unidirectional Abaca Fiber/VE Self-Healing Panels

**Figure 1:**
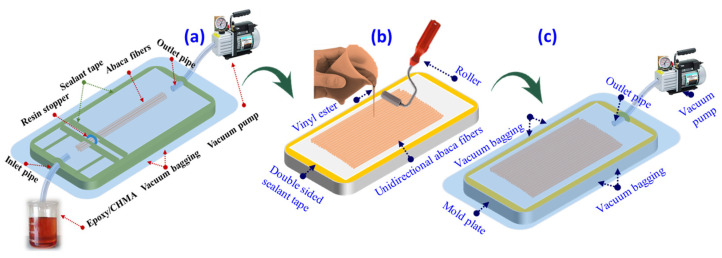
(**a**) Method of infusing healing cores into the abaca fiber lumens. (**b**,**c**) Hand lay-up method for the manufacturing of unidirectional AF/VE panels.

The hand lay-up technique was used to create two types of unidirectional AF/VE panels: empty-core abaca lumen-reinforced panels (ECA-VE) and resin-core abaca lumen-reinforced panels (RCA-VE). The chosen manufacturing process was the most efficient method for creating panels without disturbing the lumen core (healing resins). Abaca fibers were processed into two layers, each weighing 24 g and measuring roughly 30 cm in length. In a steel mold, the complete fiber lay-up was placed. Subsequently, the matrix was added, the second layer was added on top of the first, the matrix was poured, and the resin distribution was rendered uniform with a roller. A vacuum bag was used to cover the entire setup at a pressure of 0.03 MPa (see Figure 1). The setup was then placed in a composite curing oven at 60 °C for 2 h.

### 2.3. Tests and Characterization

Morphology studies were conducted using SEM (SEM; Emcrafts Cube 2, Seoul, Republic of Korea) with an ion sputter coater at 20 kV. The chemical compositions of the panels were characterized using field emission SEM-EDX mapping (FESEM-EDX; TESCAN, LYRA3XM, Turnov, Czech Republic). The thermal decomposition of the fibers, healing materials, and fibers with healing agents were characterized using a thermogravimetric analyzer (Perkin Elmer STA 6000, PerkinElmer, Inc., Waltham, MA, USA). The weight of the sample ranged from 10 to 12 mg, with a temperature of 30–700 °C at a rate of 20 °C/min under a nitrogen atmosphere. The tensile testing was conducted in accordance with ASTM D-3039 (250 mm × 25 mm) on unidirectional AF/VE self-healing panels using a universal testing machine (Model: UTM-M (RB301, UNITECH-T, R & B Inc., Daejeon, Republic of Korea), load cell: 100 kN, USA) with a cross-head speed of 2 mm/min. The tests were performed before and after partially damaging the panels with a drop-weight impact (model: RB-310 DWIT, R&B Inc., Daejeon, Republic of Korea) with a spherical indenter (d = 12.7 mm). The averages of three specimens were reported. The flexural test was conducted in accordance with ASTM D-790 (65 mm × 12.7 mm) using a universal testing machine (Model: UTM-M (RB301), load cell: 100 kN, UNITECH-T, R & B Inc., Daejeon, Republic of Korea), with a cross-head speed of 2 mm/min. The averages of three specimens were reported. The energy absorption of the self-healing VE panels was tested according to ASTM D-256 using an Izod impact testing machine (Model: QC-639 F (Cometech, Gyeonggi, Republic of Korea)) with a capacity of 22 joules.

## 3. Results

### 3.1. Structural Features of Abaca Fiber before and after Resin Infusion

**Figure 2:**
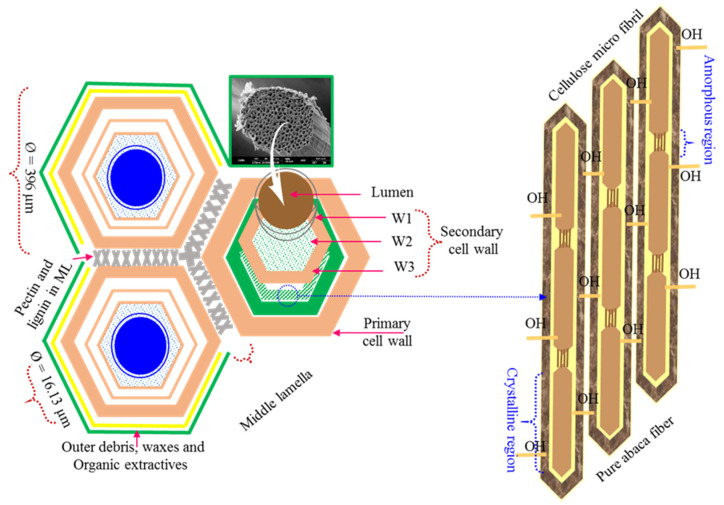
Hierarchical structure of an abaca fiber lumen and the cell wall with cellulose groups.

**Figure 3:**
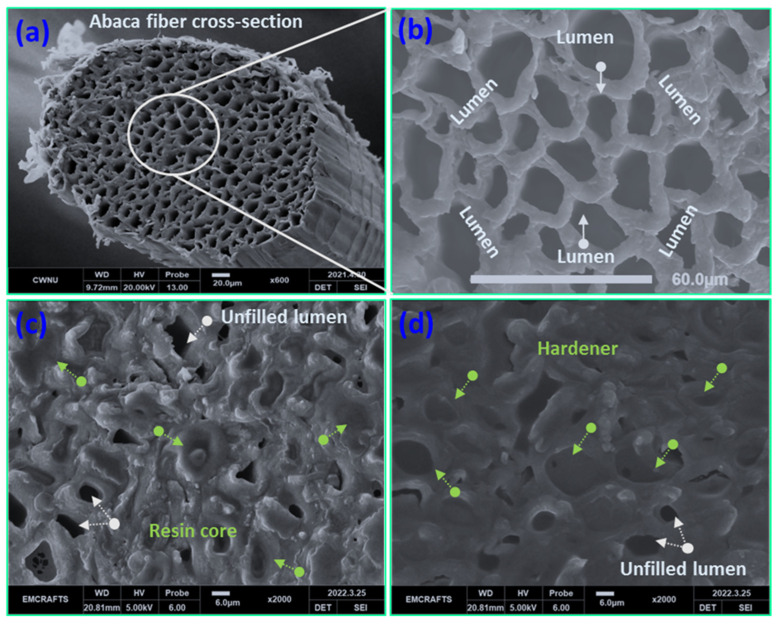
(**a**) Cross-sectional view of abaca fiber, (**b**) cross-section view of abaca fiber with empty lumens, (**c**) lumens filled with healing resin, and (**d**) lumens filled with catalyst (hardener) core.

The average diameters of technical unidirectional abaca fibers and lumen fibers were 396 μm and 16.13 μm, respectively (see Figure 2). SEM was used to conduct a typical analysis of abaca fibers before and after the injection of healing drugs into the lumens. A single abaca fiber is made up of a collection of vascular lumens that have been split into separate lumens by a cell wall, as shown in Figure 3a. The fiber’s outermost layer has a finely grooved surface that connects it to the next fibril, resembling a shield for the lumen. Abaca fibers typically have 250–400 microlumens per fiber. The structure of each empty lumen was found to vary in shape, with circles and ellipses being the most prevalent shapes, as shown in Figure 3b. A notably thick cell wall separating each lumen aids in the preservation of the liquid for an extended period of time without disruption. The core-embedded fibers were analyzed to identify the filling and un-filling lumens, following the successful infusion of the healing resin into the lumen using the VARIM procedure. The fiber lumens filled with catalyst cores and healing resin are depicted in Figure 3c,d. The unfilled individual lumen outlet side was closed or foreign particles may have entered the lumen, as evidenced by the identification of parts of the unfilled lumen. Due to the viscosity of the healing agents, it can be seen from the core-embedded figures that 95% of the lumens were filled with them. Additionally, the lumens had adequate passage for the entire duration of the experiments. Figure 3c shows the river-like pattern observed after resin infusion owing to the behavior of the healing resin compared with that of the catalyst resin. The cores were infused into the lumen for 10 min using the VARIM technique at a maximum pressure of 0.09 bar.

### 3.2. Mechanical Properties

#### 3.2.1. Tensile Strength

**Figure 4:**
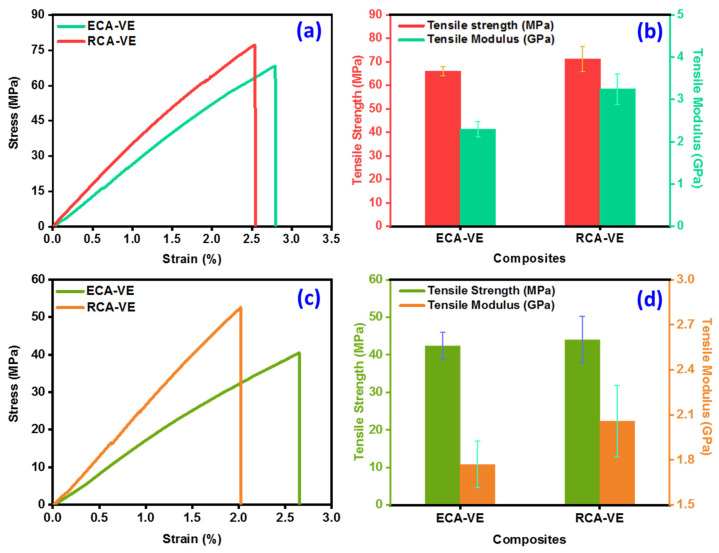
(**a**,**b**) Tensile stress–strain curves and strengths of pure and core embedded AF/VE panels. (**c**,**d**) Tensile stress–strain curves and strengths of pure and core embedded AF/VE panels (after healing).

To assess the strength of the panels under tensile loading, VE resin panels embedded with unidirectional abaca vascular lumen fibers underwent tensile tests. The initial tensile strength of the pure and core-embedded AF/VE panels was tested in two different ways. In the first instance, the tensile strengths of the healing-core-embedded abaca fiber (RCA-VE) and pure abaca fiber panels with empty lumen cores (ECA-VE) were 66.04 MPa and 71.23 MPa, respectively. ECA-VE and RCA-VE had tensile moduli of 2.29 GPa and 3.24 GPa, respectively (see Figure 4a,b). Pure and core-embedded AF/VE panels exhibited an enhanced tensile strength and modulus, at 7.85% and 41.48%, respectively. When compared to the ECA-VE panels, the tensile strength was somewhat higher. The core was contained inside the lumens of the core-embedded panels, greatly increasing the interfacial area of the fiber and the strength of the self-healing panels, which was principally responsible for the increased strength. Due to the increased area of the fiber that was engaged with the matrix, the area of the fiber had a considerable impact on this strength enhancement. The diameter of the fiber lumens significantly increased (from 385 μm to 400 μm) as a result of the healing chemicals inside them. After the fiber was infused with healing resin, this increases its interaction surface area, creating an improved matrix.

In the second instance, the core-embedded AF/VE tensile specimens were partially damaged by means of a low-velocity impact tests, taking into account a potential energy of 9.67 joules, to assess the self-healing effect of pure AF/VE. The application of potential energy can create partial damage or delamination of the healing carriers at the center of the specimens, resulting in the breakage or rupture of individual healing agents and embedded fibers in that region. Vascular fibers in the undamaged area are not destroyed; thus, they cannot spread the healing chemicals into the wounded area [33]. The healing substance seeps into the affected area after the damage has occurred. The penetration of the indenter was about 1.2 mm, and the crack’s length was estimated to be between 12 and 15 mm. In order to promote healing, the specimens were kept at room temperature for 24 h. The specimens were evaluated using a universal testing machine under tensile loading after healing for 24 h. The strengths of the healed ECA-VE and RCA-VE panels were 42.42 and 44.02 MPa, respectively (see Figure 4c,d) and the tensile moduli of the ECA-VE and RCA-VE panels were 1.77 and 2.05 GPa, respectively. Four specimens’ data were examined after the tensile tests. There was a noticeable increase in strength (3%). The healing epoxy repaired the damage and sealed the panel’s delaminated cracks. Table 1 presents the mechanical properties of the damaged and undamaged ECA-VE and RCA-VE self-healing panels.

#### 3.2.2. Flexural Strength

**Figure 5:**
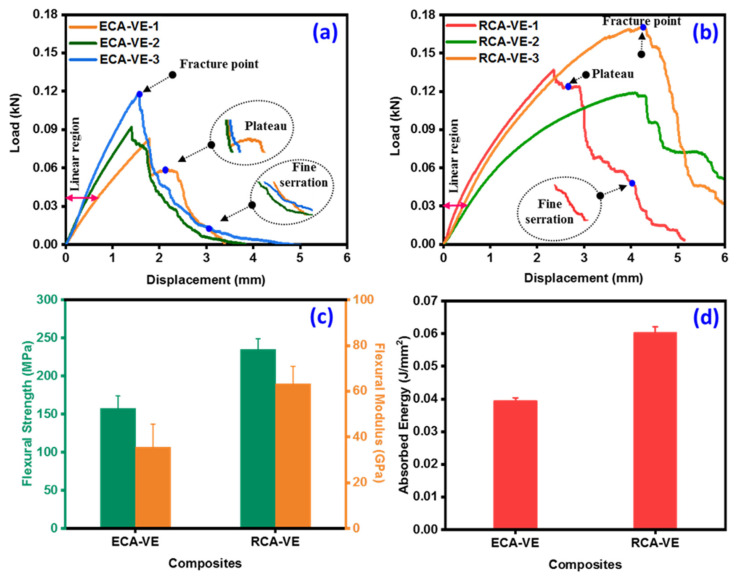
(**a**,**b**) Load–displacement curves of pure and core embedded abaca/VE panels, (**c**) flexural strength, and (**d**) Izod impact strength of abaca/VE panels.

To assess the load-bearing capacity, flexural tests was carried out on pure and healing-core-embedded AF/VE panels. Figure 5a,b show the representative load versus displacement curves of the ECA-VE and RCA-VE panels. The average load-bearing capacities of the ECA-VE and RCA-VE panels were 0.096 and 0.142 kN, respectively. The displacements for the ECA-VE and RCA-VE panels were between 3.35 to 5.0 mm and 5.1 to 8 mm, respectively. Our findings showed that the distribution of abaca vascular fibers in the resin matrix displayed a flexibility gradient commensurate with the tension condition of the outer surface of the abaca vasculature under a typical load. The curves were divided into two parts based on the flexural strain at the final fracture point. Following the plastic range, the ECA-VE and RCA-VE panels passed through the linear elastic range, before gradually reaching maximum stress. The panels displayed a plateau, then a falling trend with brief peaks that resembled fine serration (Figure 5a,b). The creation of macrocracks in the panel matrix caused a plateau, which was caused by the vascular fibers with and without healing cores carrying a constant tensile force. The debonding, fracture, and fiber pull-out that took place throughout the failure process were also reflected in the form of fine serration.

According to Figure 5c, the flexural strengths of the ECA-VE and RCA-VE panels were 157.39 MPa and 235.19 MPa, respectively. Additionally, the ECA-VE and RCA-VE panels had flexural moduli of 35.68 GPa and 63.19 GPa, respectively. In comparison to the ECA-VE panels, the RCA-VE panels’ flexural strength and modulus were 49.43% and 77.10% greater, respectively. The improved strength of the core-embedded panels can be attributed mainly to the presence of the core inside the lumens, the intrinsic toughening of vascular AF cellulose chains, and intermolecular slippage. The fiber pull-out and bridging process [34] led to toughening, which led to macroscale fracture deflection. Until the final fracture, the RCA-VE panels could bear the maximum load. This suggests that having a core inside the lumen improved fiber–matrix interaction, leading to a larger capacity for transporting loads. The abaca vascular lumen played a key role in preventing fracture propagation in the direction perpendicular to the composite, increasing the strength of the RCA-VE panel.

#### 3.2.3. Izod Impact Strength

**Figure 6:**
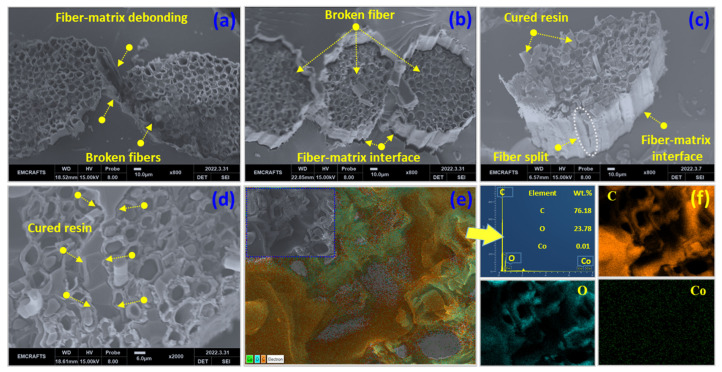
(**a**,**b**) Tensile fracture surface of pure and core-embedded AF/VE panels. (**c**) Fracture surface of core-embedded panels after Izod impact test. (**d**) Healed fracture surface after tensile test. (**e**,**f**) SEM-EDX results obtained at the self-healed region after tensile fracture.

The results of the Izod impact strength testing for the ECA-VE and RCA-VE panels are shown in Figure 5d. These findings suggest that the increased energy absorption by RCA-VE panels occurred following their recovery. The ECA-VE and RCA-VE panels absorbed energies of 0.039 J/mm^2^ and 0.060 J/mm^2^, respectively. Comparatively more energy was absorbed by the RCA-VE panels than the ECA-VE panels. The mending core’s lumens, which shifted the direction of the fracture, were thought to be responsible for this improved behavior. Compared to the empty-core reinforced (ECA-VE) panels, the vascular fiber panels with embedded cores (RCA-VE) showed greater energy absorption. This demonstrates the direct connection between the fiber lumen panels with no core implanted and those with cores. Both the ECA-VE and RCA-VE panels demonstrated a propensity for brittle fractures; cracks spread catastrophically and transversely in both panels. In the case of the final fracture, the RCA-VE panels had a greater fiber participation, pointing to ruptured and detached fibers from the matrix. The delamination and fiber pull-out, along with the absorption of a higher impact energy, confirmed these results. Figure 6c displays the fracture surface of the RCA-VE panels after 24 h of testing. Inside the fiber lumen was cured resin that was visible as resin bars. According to the fracture surface in Figure 6c, the fiber–matrix interaction was sufficient, in combination with the increased energy.

### 3.3. Fracture Morphology

The fracture morphology was observed after the final fracture in the tensile and Izod impact tests. Figure 6a,b show the tensile fracture surfaces of the ECA-VE and RCA-VE panels. As shown in Figure 6a, matrix debonding was observed between the two fibrils, and the abaca fibrils were torn out and separated from the cell walls [35]. By contrast, the fiber cells were well protected when the healing resin was inside the fiber lumens. Furthermore, the damaged lumens only served as the healing core on the damaged area, aiding multiple healing cycles. Figure 6b shows the improved fiber–matrix interface after the final fracture. The broken fibrils remained inside the matrix, providing evidence for the considerable improvement in the RCA-VE panels. However, the ECA-VE and RCA-VE panels exhibited brittle fracture morphologies after tensile fracture. The RCA-VE panels’ fracture morphology after 24 h of Izod impact testing is shown in Figure 6c. The graph demonstrates that lumens integrated with cores showed increased impact strength. The microfibrils were yanked from the cell structure after Izod impact injury. Due to the power of the rapid hit, fiber splitting was observed. The fiber cell underwent significant damage from the impact force in a specific direction along the lumen. Furthermore, the improved absorbed energy can be attributed to the cores inside the lumens, absorbing energy when a force was suddenly applied.

The tensile fracture morphology of the injured specimens after 24 h is shown in Figure 6d. The cured resin structure exhibited a river-like flow that emanated from the fracture surface. The bidirectional tensile force fracture was delayed while the cured resin left the lumens and began to mend them. SEM-EDX was used to verify the healed area and identify the chemical processes involved in the healing process. Figure 6e,f show the SEM-EDX mapping results of the healed zone. The figure shows the presence of carbon (C = 76.18), oxygen (O = 23.78), and cobalt naphthalate (Co = 0.01) in the healed area, confirming that healing took place after the damage from the drop weight impact, and was seen 24 h after the final fracturing of the self-healing panels. The core-embedded abaca lumen-reinforced resin panels maintained the highest interfacial adhesion between the fiber and the matrix, according to the fracture analysis.

### 3.4. Thermogravimetric Analysis (TGA)

**Figure 7:**
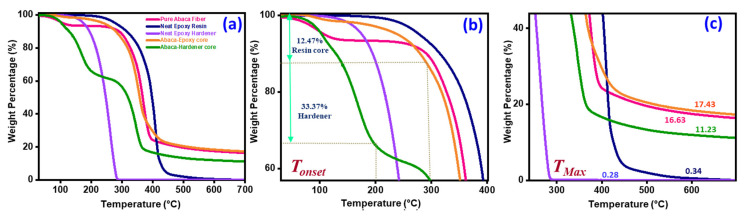
(**a**–**c**). Thermogravimetric analysis of pure abaca fiber, healing resins, and abaca fibers with healing cores.

Figure 7 displays the thermogravimetric analysis of healing resins and pure and abaca fibers. TGA was performed on the submitted samples to determine the thermal degradation temperature, the percentage of the core inside the abaca lumens, and the representative self-healing panels. The test was conducted under a nitrogen atmosphere in the temperature range of 30–700 °C at 20 °C/mm. The thermograms show different degradation temperatures in different temperature regions. The initial degradation temperature of the neat epoxy hardener was approximately 169.16 °C, as shown in Figure 7a. This was faster than that of the neat epoxy resin (285.56 °C). The early degradation (T_onset_) of the epoxy and catalyst core-embedded abaca fibers corresponds to the degradation of healing materials at the initial stage. The epoxy and catalyst cores inside the fiber lumens were 12.47% and 33.37%, respectively. The initial degradation in the pure abaca fiber started much earlier than that in the neat epoxy polymers. The faster degradation was primarily attributed to the evaporation of free and absorbed moisture, volatiles, and ash in the pure abaca fiber. Table 2 lists the individual degradation temperatures at the initial (T_onset_) and final degradation temperatures (T_max_).

The epoxy core and hardener-core-embedded abaca fiber composites were also characterized to analyze their thermal degradation. The epoxy-core-embedded abaca fibers demonstrated a trend similar to that of the pure epoxy at the initial degradation temperature owing to the decomposition of the epoxy resin at the initial stage. However, the maximum temperature of the epoxy-core-embedded abaca fibers was 425.95 °C with a final residue of 17.43 wt%. By contrast, the hardener core embedded in the abaca lumens exhibited two thermal degradation plots: the initial degradation at 103.78 °C, representing the free and absorbed moisture content in the abaca fiber, and the second degradation at 281.74 °C, corresponding to the hardener, along with the cellulose content in the fiber. After the moisture and core in the abaca fiber had degraded, ultimate degradation occurred with the abaca fiber, with a degradation peak at 375.38 °C and a residue of 11.23 wt%.

### 3.5. Self-Healing Phenomena of AF/VE Panels

**Figure 8:**
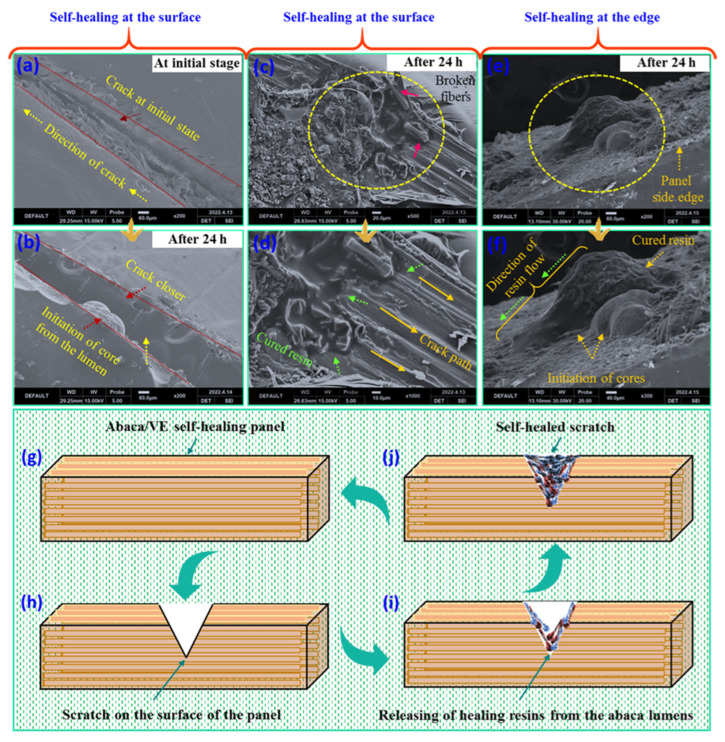
(**a**–**d**) Initial self-healing of cracked surface and after 24 h, (**e**,**f**) self-healing at the edge of the resin panels after 24 h, and (**g**–**j**) graphical representation of the self-healing mechanism of AF/VE panels.

The self-healing phenomena in unidirectional AF/VE panels were analyzed through SEM at the time of initial damage and after 24 h. Healing-core-embedded AF/VE (RCA-VE) panels were damaged with a sharp needle (outer diameter = 0.305 mm, inner diameter = 0.140, Gauge-30) on the surface. Figure 8a shows the initial damage of the panel surface with a crack width of 0.24 mm (length of the crack = ~1.93 mm, length of the healed crack = ~1.90 mm). The crack produced on the panel surface caused the core-embedded fibers in that zone to rupture. The fibers in the injured area were parallel to the crack’s route, which resulted in damage and the release of the core from the lumen. Immediately after the crack was created, the broken bulbs released healing chemicals into the crack. In order to speed up the curing process, the resin and hardener improved the exchange connections between molecules (see Figure 8g–j). According to Figure 8b, the healing substances were released from the lumens in a river-like flow, and the core’s initiation in the damaged area was spherical. After 24 h, the released healing agents had potentially closed the cracked area. Figure 8c shows the surface crack on the composite, damaging the fibers (highlighted with pink arrows) in the cracked path. Figure 8c,d show that the healing core partially flowed along the direction of the crack (crack width = 0.126 mm, crack length = 1.938 mm, and sealed crack length = 0.646 mm) and closed the crack after 24 h. The partial closing of cracks was primarily attributed to the inadequate healing materials released from the lumens, which depended on the number of damaged fibers. Moreover, a self-healing effect at the edge side of the panel was observed after 24 h. The damaged fibers released healing agents from the lumens at the edge of the panels, as shown in Figure 8e,f. The capillary action was activated to release the core materials from the lumens (Figure 8f). Moreover, the direction of the healing resin was noted. At the outer edge of the composite, there appeared to be one on top of another, because of the overlapping of the vascular abaca fibers. Figure 8i,j show a representative process of the self-healing mechanism in the composites after creating a scratch. Based on the above results, it can be concluded that the adopted natural fiber vascular lumen systems are potential healing carriers for self-healing applications in the automobile sector.

#### SEM-EDX Mapping

**Figure 9:**
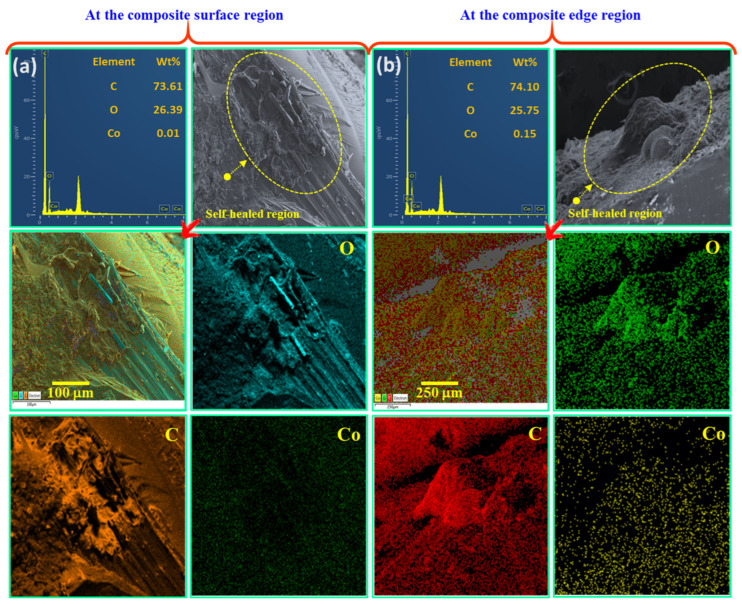
SEM images and EDX elemental maps at the (**a**) surface and (**b**) side edges of the AF/VE self-healing panels.

SEM-EDX mapping was conducted on the composite self-healing sites to determine the chemical compositions of the released healing agents. The analysis was performed on two self-healing regions in the RCA-VE panels. Figure 9a shows the carbon (C = 73.61 wt%), oxygen (O = 26.39 wt%), and cobalt naphthalate (Co = 0.01 wt%) contents. The chemical composition depended on the number of fibers damaged in the cracked zone and the amount of healing resin released from the damaged lumens. The released healing agent was cured at the edges of the composite, as shown in Figure 9b. Carbon (C = 74.10 wt%), oxygen (O = 25.75 wt%), and cobalt naphthalate (Co = 0.15 wt%) were observed. Based on the EDX data, we confirmed that the amount of released healing agents reflected the embedded core chemical composition inside the abaca lumens.

## 4. Conclusions

In this study evaluated the self-healing properties of abaca fiber lumens. The selected abaca fibers were reinforced in a vinyl ester matrix as self-healing carriers. Reinforced abaca fibers serve as a reinforcement and as healing carriers in panels. The panels were manufactured using the traditional hand-lay-up method under vacuum assistance. The lumen integrity and characteristics were studied before and after the infusion of healing materials into the lumens using SEM. Mechanical and self-healing studies were conducted using tensile tests before and after partially damaging the panels using low-velocity impact tests. The undamaged panel specimens indicated a slight improvement in tensile strength (7.85%). Furthermore, the partially damaged tensile composite specimens exhibited an improved strength of 2.92% after 24 h. The flexural strengths and load-bearing capacities of the ECA-VE and RCA-VE panels were studied. The flexural test results demonstrated that the flexural strength and modulus of the RCA-VE panels were 49.43% and 77.10%, respectively. The presence of a core bears the load in the direction normal to the composite, significantly enhancing its flexural strength and modulus. The absorbed energy of the panels was tested using the Izod impact test with 22 joules of energy. The core-embedded panels showed an improved absorbed energy of 53.84% compared with the pure composite. The improved absorbed energy was primarily attributed to the healing core, which can absorb energy during impact. Fracture analysis provided evidence for improved mechanical strength. The self-healing properties of the manufactured panel were investigated using SEM at various locations on the panel surface. The findings revealed that healing occurred in the damaged zone and that the fracture was closed by the release of the resin and catalyst. After a 24-h period, the crack with a length of 1.84 mm and a width of 0.24 mm was completely closed with healing resin. The results showed that reinforced abaca vascular lumen systems can protect against damage and extend the life of panels. These manufactured resin panels are the best alternatives to medium-strength synthetic composite panels in the automobile sector.

## Figures and Tables

**Table 1:** Mechanical properties of abaca fiber/VE self-healing panels.

Mechanical Properties	Tensile Strength (MPa)	Tensile Modulus (GPa)	Standard Deviation	Flexural Strength (MPa)	Flexural Modulus (GPa)	Standard Deviation	Izod Impact Strength (J/mm^2^)	Standard Deviation
Undamaged (ECA-VE)	66.04	2.29	1.94	157.39	35.59	16.21	0.039	0.0009
Undamaged (RCA-VE)	71.23	3.24	5.41	235.19	63.19	13.64	0.060	0.00181
Damaged (ECA-VE)	42.42	1.77	3.53	-	-	-	-	-
Damaged (RCA-VE)	44.02	2.05	6.20	-	-	-	-	-

**Table 2:** Thermal degradation temperatures and residues of pure abaca fiber, healing resins, and abaca fibers with healing cores.

Specimens	T_onset_ (°C)	T_max_ (°C)	Percentage of Core (%)	Char @ 700 (°C)
Pure abaca fiber	287.70	401.50	-	16.63
Neat Epoxy resin	285.56	445.04	-	0.34
Neat Hardener	169.16	285.56	-	0.28
Abaca-Epxy core	277.31	425.95	12.47	17.43
Abaca-Harener	103.78, 281.74	375.38	33.37	11.23

## Data Availability

There are no data available.

## References

[1] Smojver I., Ivančević D., Brezetić D. (2022). Modelling of micro-damage and intrinsic self-healing in unidirectional CFRP composite structures. Compos. Struct..

[2] Kumar Pittala R., Dhanaraju G., Satish Ben B., Avinash Ben B. (2022). Self-healing of matrix cracking and delamination damage assessment in microcapsules reinforced carbon fibre epoxy composite under flexural loading. Compos. Struct..

[3] Khan N.I., Halder S., Thomas S. (2020). Chapter 15—Self-healing fiber-reinforced polymer composites for their potential structural applications. Self-Healing Polymer-Based Systems.

[4] Mobaraki M., Ghaffari M., Mozafari M., Khan A., Jawaid M., Raveendran S.N., Asiri A. (2020). Self-healing polymers for composite structural applications. Self-Healing Composite Materials.

[5] Moniruzzaman M., Christogianni P., Kister G. (2016). Self-Healing in Epoxy Thermoset Polymer Films Triggered by UV Light. Procedia Eng..

[6] Scheiner M., Dickens T.J., Okoli O. (2016). Progress towards self-healing polymers for composite structural applications. Polymer.

[7] Venkata Chalapathi K., Cheedarala R.K., Song J.I. (2021). Synthesis of restorative microcapsules having vinyl ester healing medicine for hybrid resin/modified carbon fiber self-healing composites. J. Appl. Polym. Sci..

[8] Romero-Sabat G., Gago-Benedí E., Roa Rovira J.J., González-Gálvez D., Mateo A., Medel S., Chivite A.T. (2021). Development of a highly efficient extrinsic and autonomous self-healing polymeric system at low and ultra-low temperatures for high-performance applications. Compos. Part A Appl. Sci. Manuf..

[9] Li Y., Zhang D., Li J., Lu J., Zhang X., Gao L. (2022). Application of hierarchical bonds for construction an anti-corrosion coating with superior intrinsic self-healing function. Colloids Surf. A Physicochem. Eng. Asp..

[10] Ouarga A., Lebaz N., Tarhini M., Noukrati H., Barroug A., Elaissari A., Ben Youcef H. (2022). Towards smart self-healing coatings: Advances in micro/nano-encapsulation processes as carriers for anti-corrosion coatings development. J. Mol. Liq..

[11] Thakare A.A., Gupta T., Deewan R., Chaudhary S. (2022). Micro and macro-structural properties of waste tyre rubber fibre-reinforced bacterial self-healing mortar. Constr. Build. Mater..

[12] Venkata Prasad C., Yeriswamy H., Sudhakar P., Sudhakara P., Subha M.C.S., Chowdoji Rao K. (2018). Preparation and characterization of nanoparticle filled, mixed matrix membranes for the pervatportion dehydration of isopropyl alcohol. J. Appl. Poly. Sci..

[13] Naga Kumar C., Prabhakar M.N., Song J. (2020). Result of vascular tube design on the curative and mechanical performance of modified carbon fibers/hybrid resin self-healing composites. Polym. Compos..

[14] Li P., Du Y., Liu G., Huang P., Ding Y. (2021). Light self-healing of resin matrix composites based on pipe network carrier. Mater. Lett..

[15] Selvarajoo T., Davies R.E., Freeman B.L., Jefferson A.D. (2020). Mechanical response of a vascular self-healing cementitious material system under varying loading conditions. Constr. Build. Mater..

[16] Pietruszczak S., Przecherski P. (2021). On hydro-mechanical response of self-healing and self-sealing fractured geomaterials. Comput. Geotech..

[17] Huang H., Tang X., Xie K., Peng Q. (2021). Enhanced self-healing of irradiation defects near a Ni–graphene interface by damaged graphene: Insights from atomistic modeling. J. Phys. Chem. Solids.

[18] Duan Z., He H., Liang W., Wang Z., He L., Zhang X. (2018). Tensile, Quasistatic and Dynamic Fracture Properties of Nano-Al_2_O_3_-Modified Epoxy Resin. Materials.

[19] Mia X., Zhonga L., Wei F., Zeng L., Zhang J., Zhang D., Xu T. (2019). Fabrication of halloysite nanotubes/reduced graphene oxide hybrids for epoxy composites with improved thermal and mechanical properties. Polym. Test..

[20] Rahmani H., Eslami-Farsani R., Ebrahimnezhad-Khaljiri H. (2020). High Velocity Impact Response of Aluminum- Carbon Fibers-Epoxy Laminated Composites Toughened by Nano Silica and Zirconia. Fibers Polym..

[21] Anjabin R., Khosravi H. (2019). Property improvement of a fibrous composite using functionalized carbon nanofibers. Polym. Compos..

[22] Fan K., Wang L., Wei W., Wen F., Xu Y., Zhang X., Guan X. (2022). Multifunctional self-healing eutectogels induced by supramolecular assembly for smart conductive materials, interface lubrication and dye adsorption. Chem. Eng. J..

[23] Feng L., Yu Z., Bian Y., Wang Y., Zhao Y., Gou L. (2018). Effect of failure modes on healing behavior and multiple healing capability of self-healing polyurethanes. Constr. Build. Mater..

[24] Sudhakara K., Obi Reddy K., Venkata Prasad C., Jagadeesh D., Kim H.S., Kim B.S., Bae S.I., Song J.I. (2013). Studies on Borassus fruit and its composites with polypropylene. Compos. Res..

[25] Chalapathi K.V., Prabhakar M.N., Song J.I. (2022). Impact of Surface Treatments and Hybrid Flame Retardants on Flammability, and Thermal Performance of Bamboo Paper Composites. J. Nat. Fibers.

[26] Yang C., Zhu D., Sun C., Chen B., Li Y., Pulidindi I.N., Zheng Z., Wang X. (2021). Electrothermally responsive self-healing for carbon fiber/epoxy interphase based on Diels-Alder adducts. Compos. Sci. Technol..

[27] Ostapiuk M., Loureiro M.V., Bieniaś J., Marques A.C. (2021). Interlaminar shear strength study of Mg and carbon fiber-based hybrid laminates with self-healing microcapsules. Compos. Struct..

[28] Wang B., Yuan Z., Liu G., Zhao G., Hao W. (2021). Estimating self-healing capability of carbon fiber/epoxy composites using ultrasonic guided wave. Polym. Test..

[29] Sun Y., Liu W., Xu D., Li X., Li C. (2020). Self-healing of super hydrophobic and hierarchical surfaces for gas diffusion layer. Int. J. Hydrogen Energy.

[30] Liu K., Takagi H., Yang Z. (2013). Dependence of tensile properties of abaca fiber fragments and its unidirectional composites on the fragment height in the fiber stem. Compos. Part A Appl. Sci. Manuf..

[31] Punyamurthy R., Sampathkumar D., Ranganagowda R.P.G., Bennehalli B., Srinivasa C.V. (2017). Mechanical properties of abaca fiber reinforced polypropylene composites: Effect of chemical treatment by benzenediazonium chloride. J. King Saud Univ.-Eng. Sci..

[32] Fan W., Jin Y., Shi L., Du W., Zhou R. (2020). Transparent, eco-friendly, super-tough “living” supramolecular polymer with fast room temperature self-healability and reprocessability under visible light. Polymer.

[33] Pang J.W.C., Bond I.P. (2005). A hollow fibre reinforced polymer composite encompassing self-healing and enhanced damage visibility. Compos. Sci. Technol..

[34] Obataya E., Kitin P., Yamauchi H. (2007). Bending characteristics of bamboo (*Phyllostachys pubescens*) with respect to its fiber–foam composite structure. Wood Sci. Technol..

[35] Li Y., Ma H., Shen Y., Li Q., Zheng Z. (2015). Effects of resin inside fiber lumen on the mechanical properties of sisal fiber reinforced composites. Compos. Sci. Technol..

